# Electric-Field Control in Phosphorene-Based Heterostructures

**DOI:** 10.3390/nano12203650

**Published:** 2022-10-18

**Authors:** Calin-Andrei Pantis-Simut, Amanda Teodora Preda, Nicolae Filipoiu, Alaa Allosh, George Alexandru Nemnes

**Affiliations:** 1 Horia Hulubei National Institute for Physics and Nuclear Engineering, 077126 Magurele-Ilfov, Romania; 2 Faculty of Physics, University of Bucharest, 077125 Magurele-Ilfov, Romania; 3 Research Institute of the University of Bucharest (ICUB), Mihail Kogalniceanu Blvd 36-46, 050107 Bucharest, Romania

**Keywords:** phosphorene, graphene, hexagonal boron nitride, nanoribbon, electric-field control

## Abstract

Phosphorene is a graphene-like material with an intermediate band gap, in contrast to zero-gap graphene and large-gap dichalcogenides or hexagonal boron nitride (hBN), which makes it more suitable for nanoelectronic devices. However, inducing band-gap modulation in freestanding phosphorene nanoribbons (PNRs) is problematic, as high in-plane electric fields are necessary to close the gap. We perform here a detailed investigation concerning the substrate influence on the electric-field control exerted by an external gate, using the density functional theory–non-equilibrium Green’s functions (DFT-NEGF) framework. It is established that the interaction with a hexagonal boron nitride supporting layer significantly enhances the gap modulation. Furthermore, we address the issue of contacting the PNRs, by using conducting graphene nanoribbons embedded in the support hBN layer. Within this setup, a measurable spin polarization is achieved owing to the anti-ferromagnetic coupling between the edges of the graphene nanoribbons.

## 1. Introduction

Two-dimensional graphene-like materials have been focused on in the past few years, widening the range of potential applications, which includes nanotransistors, optoelectronic and storage devices [1,2,3,4,5]. In spite of the exceptional qualities of graphene, established as a high-mobility, ultrathin conductor, its zero gap and the known difficulty to open it remained problematic for field-effect applications. With the advent of other graphene analogues, such as hexagonal boron nitride (hBN), transition-metal dichalcogenides (TMDs), group-IV silicene and germanene and III-VI compounds, the number of potential 2D materials candidates has significantly increased. However, none of the aforementioned candidates have the expected semiconducting properties: silicene and germanene have a zero gap like graphene and inducing a gap requires additional processing, such as functionalization [6]; the hBN monolayer is almost insulating with a band gap of ∼6.47 eV [7]; and the TMDs have smaller band gaps but are still about ∼1.5 eV or larger [8,9].

Another even more promising 2D material is phosphorene, which has been isolated from black phosphorous (BP) [10]. With a smaller band gap of ∼1.5 eV as the monolayer BP and as low as ∼0.3 eV for a few-layer system [11], it is regarded as a bridging element between graphene and the TMDs. With its puckered crystalline structure, the few-layer BP structures exhibit high carrier mobilities [12] and anisotropic effects [13], which can be exploited in the design of nanoelectronic devices.

Pristine phosphorene has shown remarkable properties, and extensive theoretical and experimental investigations have been carried out. The presence of a van Hove singularity and ferromagnetic instability was demonstrated [14], while the k·p theory was employed to determine the effective g-factors, Landau levels and exciton energies [15]. Furthermore, spin–orbit coupling and spin relaxation were investigated in phosphorene, revealing potential spintronic applications [16]. Using transition metals as extrinsic doping [17], adsorption [18] or in the context of more complex point defects [19], a half-metallic behavior is induced. Furthermore, there is an increasing interest in the fabrication of phosphorene-hBN and phosphorene-graphene heterostructures, as evidenced by recent studies [20,21].

For device applications, quasi one-dimensional phosphorene nanoribbons (PNRs) can be etched or patterned from the 2D monolayer, with resulting structures in either zig-zag or armchair terminations depending on the cutting direction. It was found that pristine zig-zag phosphorene nanoribbons (zPNRs) have a metallic character, while the armchair phosphorene nanoribbons (aPNRs) have semiconducting behavior [22]. Edge passivation by hydrogen changes the metallic character of zPNRs to semiconducting, while energy gaps for both zPNRs and aPNRs decrease to the value obtained for the 2D monolayer, as their widths are increasing. Applying a lateral electric field, the band gaps of passivated zPNRs and aPNRs can be reduced and eventually closed [23]. This can be further influenced by edge modifications employing non-metallic atoms [24]. Edge functionalization by transition-metal (TM) atoms produces spin polarization, and rectification for a spin component can be achieved [25]. Doping and edge passivation play an important role in tuning the transport properties, as it is shown for aPNRs exhibiting a negative differential resistance (NRD) [26].

In this paper, we investigate by ab initio calculations the electric-field control on the electronic and transport properties of phosphorene-based one-dimensional heterostructures, where the support layer is either an hBN nanoribbon or a graphene-hBN-graphene (G-hBN-G) double junction. Stacking phosphorene on hBN or graphene has been previously analyzed for 2D monolayers [27], revealing a charge transfer and implying the tunability of the transport properties of the active phosphorene layer by hBN or graphene support layers. Our analysis outlines the role of the support hBN layer in decreasing the maximal field needed to close the band gap, complementing previous studies on the transport properties of freestanding PNRs [24,25,26]. Furthermore, contacting phosphorene is problematic as a direct interface with metals can degrade it and may create undesired Schottky barriers. We approach this issue by assembling the support layer as a G-hBN-G double junction, where the conductive zig-zag graphene nanoribbons (zGNRs) take the role of metallic contacts. In this setup, spin-polarized currents can be observed, stemming from the antiferromagnetic coupling between the edges of the zGNRs.

## 2. Structures and Methods

**Figure 1:**
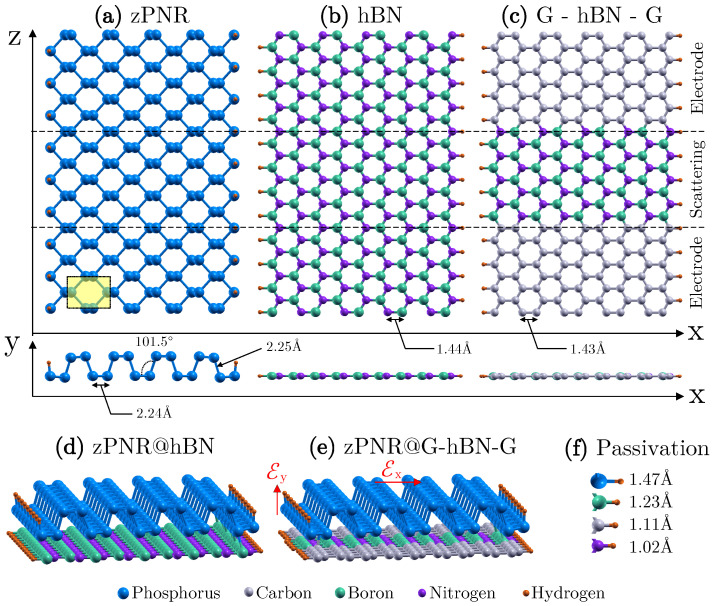
Stacked bilayer heterostructures and the assembling nanoribbons: (**a**) zig-zag phosphorene nanoribbon (zPNR); (**b**) hexagonal boron nitride (hBN) nanoribbon; (**c**) graphene-hBN-graphene (G-hBN-G) double junction; (**d**) heterojunction comprising the zPNR on top of the hBN nanoribbon (zPNR@hBN); (**e**) heterojunction comprising the zPNR and the G-hBN-G double junction (zPNR@G-hBN-G). External electric fields can be applied either in-plane ($E_{x}$) or perpendicular to the zPNR ($E_{y}$). (**f**) Bond lengths corresponding to passivating hydrogen atoms.

The stacked heterostructures are comprised of a zPNR (Figure 1a) as the active transport layer and a support layer that has two configurations: (i) hBN nanoribbon (Figure 1b) and (ii) G-hBN-G double junction (Figure 1c), as a zGNR with an embedded hBN segment. We shall denote these two structures by zPNR@hBN and zPNR@G-hBN-G, respectively. The support layers are hydrogen passivated which makes them chemically inert. The hBN layer is much less conductive compared to phosphorene due to the significantly larger gap, so that, in the zPNR@hBN configuration, the charge transport occurs practically through the zPNR. However, the interaction between the two layers influences the charge distribution and the field-effect control. On the other hand, hBN and graphene have very similar lattice constants, with a mismatch of ∼2%, which makes them ideal for assembling heterojunctions. In the zPNR@G-hBN-G configuration, the semi-infinite zGNRs take the role of the metallic contacts. The heterostructures are depicted in Figure 1d,e, along with the respective building blocks. The passivation bond lengths are indicated in Figure 1f.

The translation invariant system zPNR@hBN is reproduced in a supercell approach, containing 3 units of zPNR (54P + 6H) matching well 4 units of hBN (72BN + 8H) along the transport direction. Similarly, the supercell containing zPNR stacked on graphene is constructed, given the small mismatch between the graphene and phosphorene supercells.

The ab initio calculations are performed at DFT level, implemented in the SIESTA package [28]. A set of strictly localized numeric atomic orbitals is used as a basis set, which allows the linear scaling of the computational time with the system size. The solution of the Kohn–Sham equations is determined within the local density approximation (LDA), with the parametrization of the exchange–correlation functional proposed by Ceperley and Alder [29]. Troullier–Martins norm-conserving pseudopotentials were employed to describe the valence electrons [30]. We used a single-ζ polarized basis and the cutoff radius which adjusts the orbital confinement is set by an energy shift of 275 meV. The mesh cutoff was chosen at 300 Ry, which sets the real-space grid, and the convergence tolerance of the density matrix was fixed at $10^{-4}$. The Monkhorst–Pack scheme used in the sampling of the Brillouin zone is 1×1×3 in the semi-infinite electrodes. Relaxations are performed until the forces are less than 0.04 eV/Å. The equilibrium distance between the layers has been found at ∼2.5 Å. Subsequently, the band structures are determined for the periodic systems and the optimized atomic configurations are further used in NEGF-based transport calculations.

For the transport properties of the phosphorene-based heterostructures, we employ the non-equilibrium Green’s functions (NEGF) method implemented in the TRANSIESTA code [31]. Using NEGF, one obtains the spin-dependent transmission T(E) and spin-polarized currents, considering elastic scattering. The transmission function is calculated using the relation:

(1) $$T\left(E\right)=\mathrm{Tr}\left[G^{r}\left(E\right)\Gamma_{L}\left(E\right)G^{a}\left(E\right)\Gamma_{R}\left(E\right)\right],$$

where the trace sums over the central region basis functions, $G^{r/a}$ are the retarded/advanced Green’s functions and $\Gamma_{R,L}\left(E\right)$ matrices are calculated in terms of the lead self-energies, describing the coupling between the central region and the contacts.

The spin-dependent current is given by

(2) $$I_{\sigma }=\frac{e}{h}\int dET\left(E\right)\left[f_{\mathrm{FD}}\left(E;\mu_{R}\right)-f_{\mathrm{FD}}\left(E;\mu_{L}\right)\right],$$

where σ accounts for the spin channel σ=↑,↓. The polarization of the spin current is:

(3) $$P=\frac{I_{↑}-I_{↓}}{I_{↑}+I_{↓}}.$$

One method to gain better insight into the transport of currents at the nanoscale is the visualization of the so-called *atomic currents* and *bond currents*. To this end, we used SISL [32] for the post-processing of the data, a Python package that was developed in order to manipulate input and output files from SIESTA and TRANSIESTA.

Local currents are useful to analyze possible correlations between a particular molecular or atomistic property and its role in the charge transport within the whole system. For this reason, bond currents are also referred to as “transmission pathways” [33].

In TBtrans, the orbital currents are defined in terms of the spectral density matrix, and they are implemented using the following the equation:

(4) $$J_{\alpha \beta }=i\left[H_{\beta \alpha }A_{\alpha \beta }-H_{\alpha \beta }A_{\beta \alpha }\right],$$

where α, β are orbital indices and the factor e/ħ was left out. In order to obtain the bond currents, one simply sums over all the orbital indices

(5) $$J_{\nu \mu }=\sum_{\alpha \in O_{\nu }}\sum_{\beta \in O_{\mu }}J_{\alpha \beta },$$

where $O_{\nu }$ and $O_{\mu }$ are the orbital sets of atoms ν and μ, respectively.

Due to the continuity equation, a sum of the bond currents that cross a definite surface separating the originating electrode from the rest of the device will be equal to the total current [34,35].

In order to capture the effects of the applied electric field, we also used “atomic currents” as a tool for visualization. The atomic current is a scalar quantity which displays how much current is redistributed through each atom [32]:

(6) $$J_{\nu }=\frac{1}{2}\sum_{\mu }\left|\sum_{\alpha \in O_{\nu }}\sum_{\beta \in O_{\mu }}J_{\alpha \beta }\right|.$$

## 3. Results and Discussion

**Figure 2:**
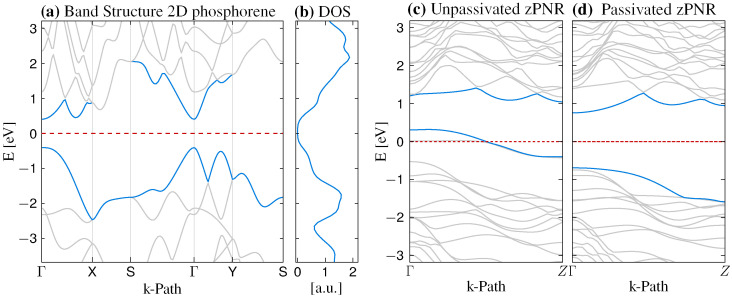
(**a**) Band structure of 2D phosphorene along a representative **k**-path, Γ-X-S-Γ-Y-S. (**b**) Density of states (DOS) for the 2D phosphorene structure. (**c**) Band structure along the transport direction, Γ-Z, for a hydrogen-passivated zPNR. (**d**) Similar to (**c**), the band structure of non-passivated zPNR is represented. The Fermi levels are marked by dashed lines.

We start our analysis by calibrating the DFT calculations on the pristine 2D phosphorene and freestanding zPNRs. In this way, we recover some of the previously reported behaviors. The electronic properties are illustrated in Figure 2, which shows band structures for both types of systems. In Figure 2a, the band structure for bulk 2D phosphorene is depicted along a representative **k**-path, evidencing the direct band gap of ∼0.82 eV, found at the Γ point. Even though the band gap is underestimated compared to the experimental value, this is expected within the LDA approximation, as for earlier GGA-based calculations [23]. The corresponding density of the states associated to the pristine 2D phosphorene is displayed in Figure 2b. Concerning the nanoribbon systems, the band structures of zPNRs with and without hydrogen passivation are illustrated in Figure 2c,d, respectively. The passivated structure exhibits a semiconductor-like behavior, with a band gap around 1.42 eV, larger than the 2D monolayer due to quantum confinement effects. However, if the passivation is removed, a metallic-like character is obtained, with two bands crossing the Fermi level. These results are consistent with other reports on the electronic structure of PNRs [24], and different passivating species can further modify the energy gap.

**Figure 3:**
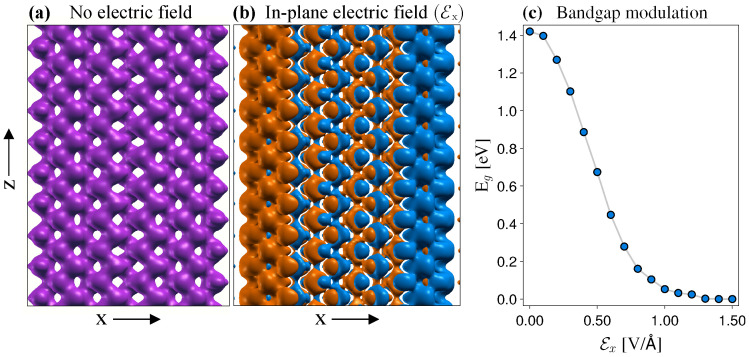
Influence of the in-plane electric field ($E_{x}$) on the charge distribution and on the band-gap energy ($E_{g}$) of zPNRs. (**a**) Charge density of the freestanding zPNR with no electric field applied. (**b**) Charge density difference, Δρ(x,z), for $E_{x}=0.5$ V/Å. (**c**) Band-gap modulation of freestanding zPNR as function of $E_{x}$.

As opposed to zGNRs, which have the charge localized mostly along the two edges, for zPNRs, the charge is more evenly distributed, as depicted in Figure 3a. This behavior is further translated in the current flow pattern, which is more localized along the central axis of the zPNR, in contrast to the established edge transport in zGNRs, as pointed out in a subsequent discussion. However, applying an in-plane electric field along the *x*-direction, of magnitude $E_{x}$, a charge imbalance occurs between the two edges, described by $\Delta \rho =\rho \left(E_{x}\right)-\rho_{0}$, where $\rho (E_{x})$ and $\rho_{0}$ are the valence charge densities with and without the applied electric field. Figure 3b shows the spatial distribution of Δρ(x,z), which exhibits large magnitudes at the two edges and decreases toward the zPNR median with alternating signs. This has implications in the charge transport, as the current in ideal zPNRs will flow predominantly within the vicinity of one of the two edges. The in-plane electric field reduces the band gap, which eventually closes at a quite large value, reaching a metallic-like behavior, as can be seen from Figure 3c. As shown in Refs. [23,24], the gap closure is influenced by the lateral width of the PNR as well as the type of passivation. However, the relatively large value necessary for the electric field is problematic in practical applications and requires further attention.

**Figure 4:**
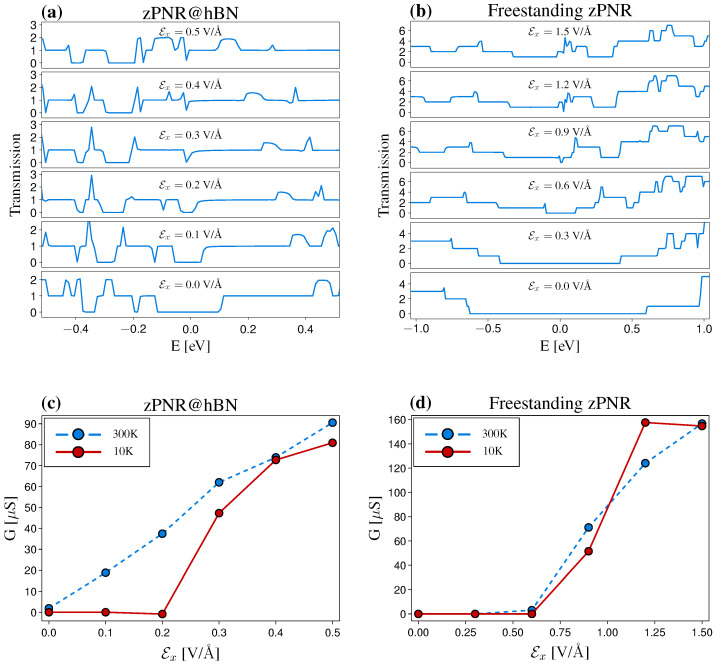
Transport properties of ideal zPNR@hBN and freestanding zPNR systems. (**a**,**b**) Transmission functions for different in-plane electric fields, $E_{x}$. The large energy gap of the freestanding zPNR is reduced by the interaction with the support layer in the zPNR@hBN bilayer structure, leading to a significantly smaller electric field necessary to close the gap ($E_{x}\approx 0.3$ V/Å). (**c**,**d**) The conductances vs. $E_{x}$ evaluated at temperatures 10 and 300 K reflect the different gap magnitudes.

In the following, we investigate the influence of the support layers on the electronic and transport properties of zPNRs, in the context of double-layer heterostructures. First, we consider the zPNR@hBN system and analyze the ideal transmission for a sequence of $E_{\mathrm{field}}$ values and compare it with the freestanding PNR. Figure 4 shows the systematic reduction in the band gap for both systems, as the in-plane electric field $E_{x}$ is increased. In the zPNR@hBN configuration, the band gap is closed at a significantly smaller $E_{x}$, in the range of 0.2–0.3 V/Å, as compared to the freestanding nanoribbon, where the gap closure occurs at $E_{x}\approx 1$ eV/Å. Consequently, the conductance, $G=2e^{2}/h\int dE\left(\partial f_{\mathrm{FD}}/\partial E\right)T\left(E\right)$, evaluated in the limit of low temperatures (10 K) and at room temperature (300 K), is sharply increasing for the heterostructure at electric fields below 0.3 V/Å, whereas for the freestanding PNR, it is limited by the poor reduction in a large gap. From the experimental point of view, the gap reduction is significant, bringing the maximum values of the electric fields in a feasible range [36,37].

**Figure 5:**
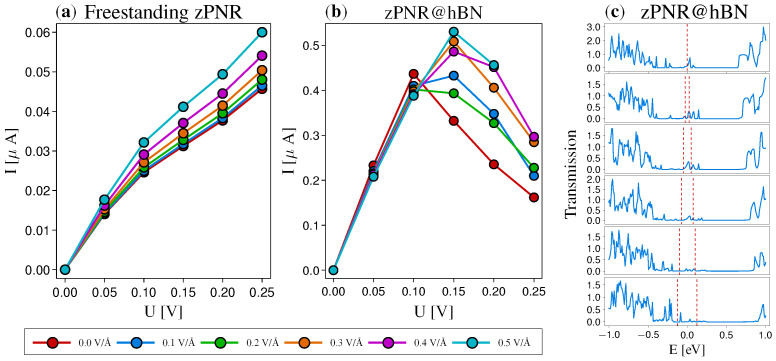
*I*–*V* characteristics for (**a**) freestanding zPNR and (**b**) zPNR@hBN for different values of the in-plane electric field, $E_{x}$, with metallic contacts emulated by non-passivated zPNRs. (**c**) Transmission functions for the zPNR@hBN systems, evidencing the bias windows (red dashed lines) for *U* in the range 0–0.25 V in steps of 0.05 V (top to down), for zero-electric field. The steady increase in the freestanding zPNR is in contrast to the high current modulation and NRD effects observed for zPNR@hBN, increasing the bias beyond 0.1 V. At large biases, the widths of the resonant energy levels in the bias window tend to decrease, reducing the current.

The analysis of the non-linear bias regime reveals further differences between the two systems, as described in Figure 5. First, the implementation of proper metallic contacts should be addressed, and one possible option is to control the edge passivation. In particular, no passivation results in the metallic behavior of zPNRs, as discussed before. Therefore, using passivated and unpassivated zPNR segments, one can assemble the device structure with contacts and an active region. On one hand, the pristine freestanding zPNR exhibits a steady increase in the current with the applied bias. In correspondence with Figure 3b, the *I*–*V* characteristics present little modulation even at high electric fields. On the other hand, this is in contrast to the zPNR@hBN system, which presents larger current magnitudes, higher modulation and, in addition, a negative differential resistance (NDR) behavior for biases *U* > 0.10 V. This can be explained based on the transmission function in the bias window [−U/2,+U/2]. At large biases, the widths of the resonant energy levels in the bias window tend to decrease, reducing the current. This can be likely assigned to enhanced reflections due to the larger potential variation under bias. On the other hand, for the freestanding zPNR, in the same range of biases (0–0.25 V), the rather insulating properties lead to a significantly smaller current, without NDR effects being present.

Although in this setup metallic-like contacts can be implemented, a controlled passivation is quite hard to achieve in practice. Because metallic contacts have certain drawbacks, such as high chemical reactivity, undesired Schottky barriers at the interfaces with the semiconducting active region, and pose further processing difficulties, we investigate here the possibility of using zGNRs as alternatives. Structurally, these can be easily embedded in the support layer, as hBN and graphene have roughly the same lattice constants. The zPNR@G-hBN-G heterostructure described in Figure 1e would benefit from the highly conductive zGNRs as the contact and, in addition, their intrinsic spin polarization can be further exploited to induce spin-polarized currents in the zPNR@G-hBN-G assembly. This has the advantage that no further functionalization of the zPNRs is needed in order to determine a certain magnetic behavior, which is usually achieved by extrinsic magnetic impurities, TMs in particular.

**Figure 6:**
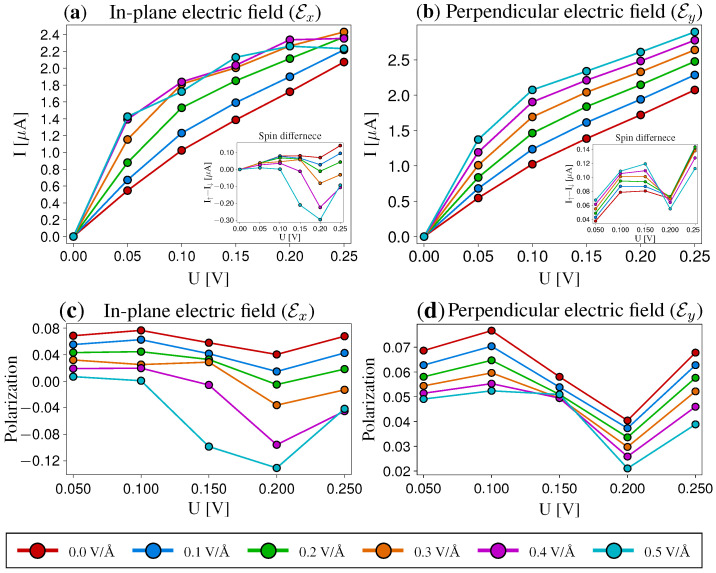
*I*–*V* characteristics (**a**,**b**) and spin polarization (**c**,**d**) for systems with *in-plane*($E_{x}$) and *perpendicular*($E_{y}$) electric fields. The modulation of the charge currents becomes similar for both electric-field orientations. The insets show the net spin currents. In addition, a spin polarization switching is found around an in-plane electric-field value $E_{x}\approx \;$0.2 V/Å at a bias U=0.2 V.

The zPNR@G-hBN-G reveals further interesting aspects concerning spin-polarized currents. Although the zPNRs alone do not present magnetic ordering, spin-polarized currents may be induced due to the underlying zGNRs, which are known for their antiferromagnetic coupling between the two edges. The relatively small interaction between the two layers, however, has two additional qualitatively significant effects, that can be observed: (i) an overall polarization of the spin currents and (ii) a modulation of the current by a perpendicular electric field ($E_{y}$). The *I*–*V* characteristics and the corresponding polarizations are depicted for the in-plane ($E_{x}$) and perpendicular ($E_{y}$) electric fields in Figure 6. In the bias range of 0–0.25 V, a considerable modulation is obtained for both orientations of the electric field, of roughly similar magnitude, as can be seen from Figure 6a,b. The currents are evaluated at 0 K. Knowing that for the pristine zPNR a perpendicular electric field does not induce any variation in the conductance, the present results indicate that bilayer phosphorene-based heterostructures offer more design options, as top gates can be used instead of lateral gates. Moreover, the magnitude of the currents is larger than in the case of the zPNR@hBN system, which can be explained by the large conductivity of the zGNRs.

The other significant effect concerns the induced spin-polarized currents without employing any extrinsic magnetic impurities. Typically, a relatively large spin polarization can be observed in quasi-1D nanoribbons of different graphene-like materials, including phosphorene, with embedded TM atoms [25,38,39,40]. However, including TMs can present deficiencies concerning the further processing of the nanoribbons, and clustering effects of the TM atoms can occur. Therefore, using an intrinsic spintronic effect, like the one found in zGNRs, could be exploited in other graphene-like materials as in the current zPNR@G-hBN-G heterostructure. The NEGF-based calculations presented in Figure 6c indicate a detectable spin polarization of ∼12%, found for applied in-plane electric fields, $E_{x}$, and spin current switching can be also evidenced in the range of 0.1–0.2 V for the highest electric fields considered. For perpendicular electric fields, $E_{y}$, under similar bias conditions, the spin polarization is smaller, reaching ∼8%, as it is shown in Figure 6d. However, these results are indicative of potential spintronic effects which can be exploited, and their magnitude can be further influenced by other factors, such as the width of the zGNR, e.g., thinner nanoribbons have a more pronounced spin polarization.

**Figure 7:**
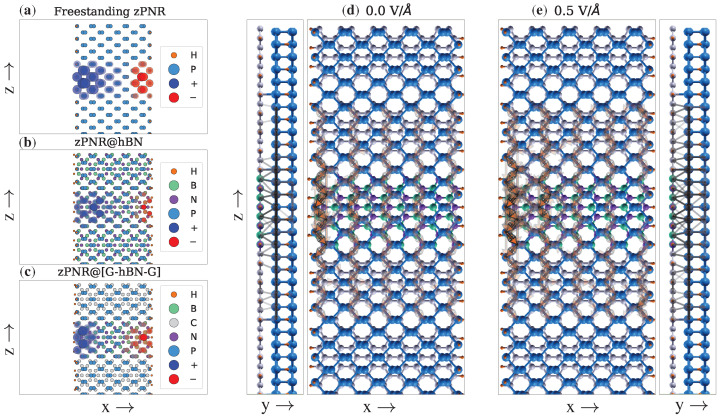
Atomic current distributions for (**a**) freestanding zPNR, (**b**) zPNR@hBN and (**c**) zPNR@G-hBN-G heterostructures, for $E_{x}=0.5$ V/Å, integrated in the [0,3] eV interval. Bond current distribution for the zPNR@G-hBN-G heterostructure with an extended central region, integrated on the [0,1.5] eV interval, for $E_{x}=0$ (**d**) and $E_{x}=0.5$ V/Å (**e**). Top and side views are indicated, outlining the current flow patterns.

A qualitative understanding of the rather intricate transport properties in the external field can be achieved using the atomic current and bond current distributions. Figure 7a–c show the atomic currents in the freestanding zPNR and in the active region of the zPNR@hBN and zPNR@G-hBN-G heterostructures. The in-plane electric field drives the mobile charge toward one of the two edges, locally enhancing the coupling between the two layers. This is directly reflected in the transmission pathways, which become spatially localized. To observe more clearly the differences, we plotted the atomic currents for zero field as the reference and the current differences obtained for $E_{y}=0.5$ V/Å. In the active region, the most significant part of the current flows through the zPNRs segments, as hBN is highly insulating. However, with the onset of the electric field, larger atomic currents become visible on the hBN underlying segment, being localized near the edge where the charge accumulation occurs. Furthermore, the bonds currents illustrate more details of the flow patterns in the zPNR@G-hBN-G structure, as shown in Figure 7d,e. Here, larger GNRs segments have been included in the central region in order to observe the influence of the G-hBN-G double junction. The zGNRs induce a more uniform distribution of currents in the overlaying zPNRs segments, while in the central region, controlled by the hBN, the currents are mostly distributed along one edge. Here, the field effect is stronger, as opposed to the regions controlled by the zGNRs, where a more effective screening takes place.

## 4. Conclusions

The charge and spin transport properties of phosphorene-based heterostructures have been investigated using ab initio NEGF-DFT calculations. We addressed three main issues, which are typically encountered in the design of field-effect applications: (i) the current modulation with in-plane and perpendicular electric fields, (ii) contact design using zGNRs and (iii) intrinsic spintronic effects. The analysis outlines the role of the underlying support layer, which is an hBN nanoribbon or the G-hBN-G double junction, by comparing the transport properties in these heterostructures with the freestanding zPNR.

The current modulation of passivated zPNRs is known to be problematic as large electric fields with in-plane orientation are required to modify the band gap. In contrast, the influence of the support layer significantly lowers the maximum electric-field value needed to close the gap. Moreover, perpendicular electric fields are shown to have a significant effect on the current modulation, unlike pristine zPNRs.

Because the realization of metallic contacts is challenging from both theoretical and practical perspectives, we explore the contact design in greater detail. The simple observation that unpassivated zPNRs have metallic character points to engineer edge passivation to influence the conduction properties. However, this is less practical, as passivation control is rather difficult, as well as subsequently retaining the system’s stability. Instead, we proposed a double-junction support layer, G-hBN-G, where the zGNRs have the role of conductive elements. The rather insulating hBN limits the current in the support layer, while the zPNR remains as the active element.

The zGNRs also have an active role in establishing spintronic effects. Originating from the antiferromagnetic spin distribution along the two edges of pristine zGNRs, spin-filtering effects can emerge when an asymmetry is introduced. The structural asymmetry introduced by the different terminations of the hBN segment, as well as the in-plane electric field, concur to a small but detectable spin polarization. This indicates the possibility to obtain a spin polarization, without employing extrinsic magnetic impurities.

To conclude, these investigations show that phosphorene-based heterostructures have great potential for field-effect and spintronic applications, outlining the better tunability compared to freestanding PNRs. This brings into attention the further potential of graphene-like materials and new design strategies for establishing efficient metallic-like contacts.

## Abbreviations

The following abbreviations are used in this manuscript:

BP	black phosphorous
DFT	density functional theory
hBN	hexagonal boron nitride
NEGF	non-equilibrium Green’s functions
NRD	negative differential resistance
TM	transition metal
zPNR	zig-zag phosphorene nanoribbon
zGNR	zig-zag graphene nanoribbon
